# Reply to Bonomo and Hujer, “Enhancing clinical utility of AI-based antimicrobial resistance models: a perspective”

**DOI:** 10.1128/mbio.01166-26

**Published:** 2026-06-17

**Authors:** Juan S. Inda-Díaz, Erik Kristiansson

**Affiliations:** 1 Centre for Microbiome Research, School of Biomedical Sciences, Queensland University of Technology, Translational Research Institute 373031, Woolloongabba, Australia; 2 Department of Mathematical Sciences, Chalmers University of Technology and University of Gothenburg 166440, Gothenburg, Sweden; 3 Centre for Antibiotic Resistance Research in Gothenburg (CARe), University of Gothenburg 3570, Gothenburg, Sweden; Instituto de Biologia Molecular y Celular de Rosario, Rosario, Santa Fe, Argentina

## REPLY

We thank Dr. Bonomo and Dr. Hujer for their thoughtful and insightful comments on our article, “Confidence-based prediction of antibiotic resistance at the patient level” (1, 2). We appreciate their recognition of the study’s technical advancement and the scale of the data used to train our AI model, containing millions of AST results from more than 30 European countries.

Major challenges must be addressed before AI-based methods for guiding the treatment of infections caused by antibiotic-resistant bacteria can be widely deployed in clinical practice. The points raised by Bonomo and Hujer, especially the lack of “real-world validation” and issues with data quality and consistency, are important and relevant culprits that today are not properly addressed by our and many other AI-based methods. In fact, the lack of evidence demonstrating benefits for patients and health care providers is a general issue for medical AI as a whole, not limited to infectious diseases (3).

The development of AI-based methods ready for clinical deployment is highly dependent on relevant data that is of sufficient size and quality. In Inda-Diaz et al., we resorted to surveillance data (from the European Surveillance System TESSy), which are comprehensive but (i) are collected from highly selected populations (invasive infections), (ii) are inconsistently generated with varying methods for AST and breakpoint definitions, (iii) have low coverage of many pathogens, (iv) do not include genotypic markers, and (v) have only limited patient metadata. Even if we demonstrate in Inda-Diaz et al. that these data enable the development of a high-performance AI method for selected pathogens and antibiotics, the data still entail considerable limitations, as correctly pointed out by Bonomo and Hujer. It should, however, be noted that our proposed transformer-based framework can incorporate information on the AST methods used, more detailed patient metadata, including electronic health records, and the presence of genotypic markers, including both resistance genes and chromosomal mutations. However, there are no data sets available for training and validation of such an AI model.

The lack of proper data is a major limitation for the development, evaluation, and deployment of AI-based methods for microbial infections (4). We argue that efforts to compile new clinically relevant data sets are crucial to moving beyond Inda-Diaz et al. and to introducing AI into routine diagnostics. Here, large volumes of “real-world” phenotypic and genotypic data reflecting clinically relevant populations will be key to covering a broad range of pathogens and infections. Also, clinical data describing patient and pathogen diversity, preferably at a global scale, will be vital to ensure that the AI-based methods are sufficiently generalizable and safely adoptable across different health care settings, regardless of catchment populations and measurement technologies. Improved data quality and quantity will also further enhance performance and reduce the impact of class imbalance, which, as noted by Bonomo and Hujer, was an issue in the data used by Inda-Diaz et al.

Finally, we wholeheartedly agree with Bonomo and Hujer that AI, at least in its current form, should be used to complement, not replace, clinicians’ medical experience and expertise. We would, however, like to emphasize that AI-based methods, such as the one presented in Inda-Diaz et al., can summarize empirical data from millions of previous infections. When combined with uncertainty estimation, such methods not only provide estimates of the most likely resistance phenotype but also assess the strength of the current diagnostic evidence from historical records, which may guide decisions about the need for further testing.
